# Immune cells and immune cell-targeted therapy in chronic pancreatitis

**DOI:** 10.3389/fonc.2023.1151103

**Published:** 2023-03-09

**Authors:** Yu Zhang, Wen-Qing Zhang, Xin-Yuan Liu, Qi Zhang, Tao Mao, Xiao-Yu Li

**Affiliations:** Department of Gastroenterology, The Affiliated Hospital of Qingdao University, Qingdao, China

**Keywords:** chronic pancreatitis, pancreatic stellate cells, immunotherapy, T cell, macrophage

## Abstract

In recent years, studies have attempted to understand the immune cells and mechanisms underlying the pathogenesis of chronic pancreatitis (CP) by constructing a model of CP. Based on these studies, the innate immune response is a key factor in disease pathogenesis and inflammation severity. Novel mechanisms of crosstalk between immune and non-immune pancreatic cells, such as pancreatic stellate cells (PSC), have also been explored. Immune cells, immune responses, and signaling pathways in CP are important factors in the development and progression of pancreatitis. Based on these mechanisms, targeted therapy may provide a feasible scheme to stop or reverse the progression of the disease in the future and provide a new direction for the treatment of CP. This review summarizes the recent advances in research on immune mechanisms in CP and the new advances in treatment based on these mechanisms.

## 1 Introduction

Pancreatitis is the main inflammatory disease of the gastrointestinal tract (1–4), Chronic pancreatitis(CP) is one of the classifications of pancreatitis. CP is a repeated or persistent injury of the acinar and tubules in the pancreatic parenchyma caused by alcohol consumption autoimmune, genetic, and other factors, resulting in atrophy or disappearance of acinar and pancreatic islet cells, which are eventually replaced by fibrotic tissues, resulting in varying degrees of pancreatic/exocrine dysfunction. It is defined as a pathological fiber inflammatory syndrome of the pancreas (3). The pathophysiology of CP is complex, including acinar cell damage, acinar stress response, ductal dysfunction, persistent or altered inflammation, and/or neuroimmune crosstalk; however, these mechanisms are not well understood (3). Acute pancreatitis(AP), recurrent AP, and CP are generally considered a continuum of disease (5, 6). Approximately 20% of patients with AP develop recurrent AP, and approximately 35% of recurrent AP progress to CP (7, 8). Existing studies have found that the pathogenesis of chronic pancreatitis is closely related to a variety of immune cells. The function of immune cells, the interaction between immune cells and the interaction between immune cells and pancreatic related cells will affect the progression of chronic pancreatitis (9, 10).

CP is characterized by persistent inflammation of the pancreas with progressive loss of endocrine and exocrine compartments due to atrophic and/or fibrotic tissue replacement (11). The functional consequences include recurrent or persistent abdominal pain, diabetes (endocrine insufficiency), and dyspepsia (exocrine insufficiency) (3). Current treatments for CP aim to control pain and address exocrine and endocrine dysfunction (12). With the discovery of mechanisms related to immune and pancreatic stellate cells in chronic pancreatitis (9, 13), CP therapies based on these cells have emerged as potential targets for the treatment and prevention of pancreatic inflammation and fibrosis.

In this review, we discuss the role of various immune cell populations and pancreatic stellate cell populations in CP, as well as current advances in targeted therapies based on different cell populations.

## 2 Occurrence of CP

CP is a kind of autopathological fibrotic inflammatory syndrome in which the pancreatic parenchyma is repeatedly damaged and stressed under the action of gene susceptibility, external environmental factors and other risk factors, and the pancreatic tissue undergoes continuous and gradual development. There are many causative factors of CP, and it is widely believed that alcoholism, smoking, and genetic susceptibility are the main causative factors, and other factors such as hyperlipidemia, hypercalcemia, autoimmune diseases, biliary diseases, pancreatic trauma or surgical trauma, and pancreatic duct stenosis caused by acute pancreatitis have been reported. Experts at home and abroad generally believe that alcoholism is the main risk factor for CP (14, 15). An epidemiological survey of high-risk factors for large-scale chronic pancreatitis based on the population in China found that the main causative factors of chronic pancreatitis were alcoholism (35.11%) and biliary stones(34.36%), while other factors included genetic susceptibility (7.22%) and idiopathic(12.9%) (16). However, a recent multicenter epidemiological survey in the US concluded that smoking was the most common cause of chronic pancreatitis (59%), followed by alcohol abuse (53%), pancreatic duct obstruction (19%), and hyperlipidemia (13%) (17). Recently, Shounak and Chari combined treatment guidelines and recent research advances in several countries to classify chronic pancreatitis into four broad categories according to etiology and pathophysiology: chronic calcific pancreatitis, chronic obstructive pancreatitis, hormone-sensitive pancreatitis (autoimmune pancreatitis), and idiopathic chronic pancreatitis (14).

**Figure 1:**
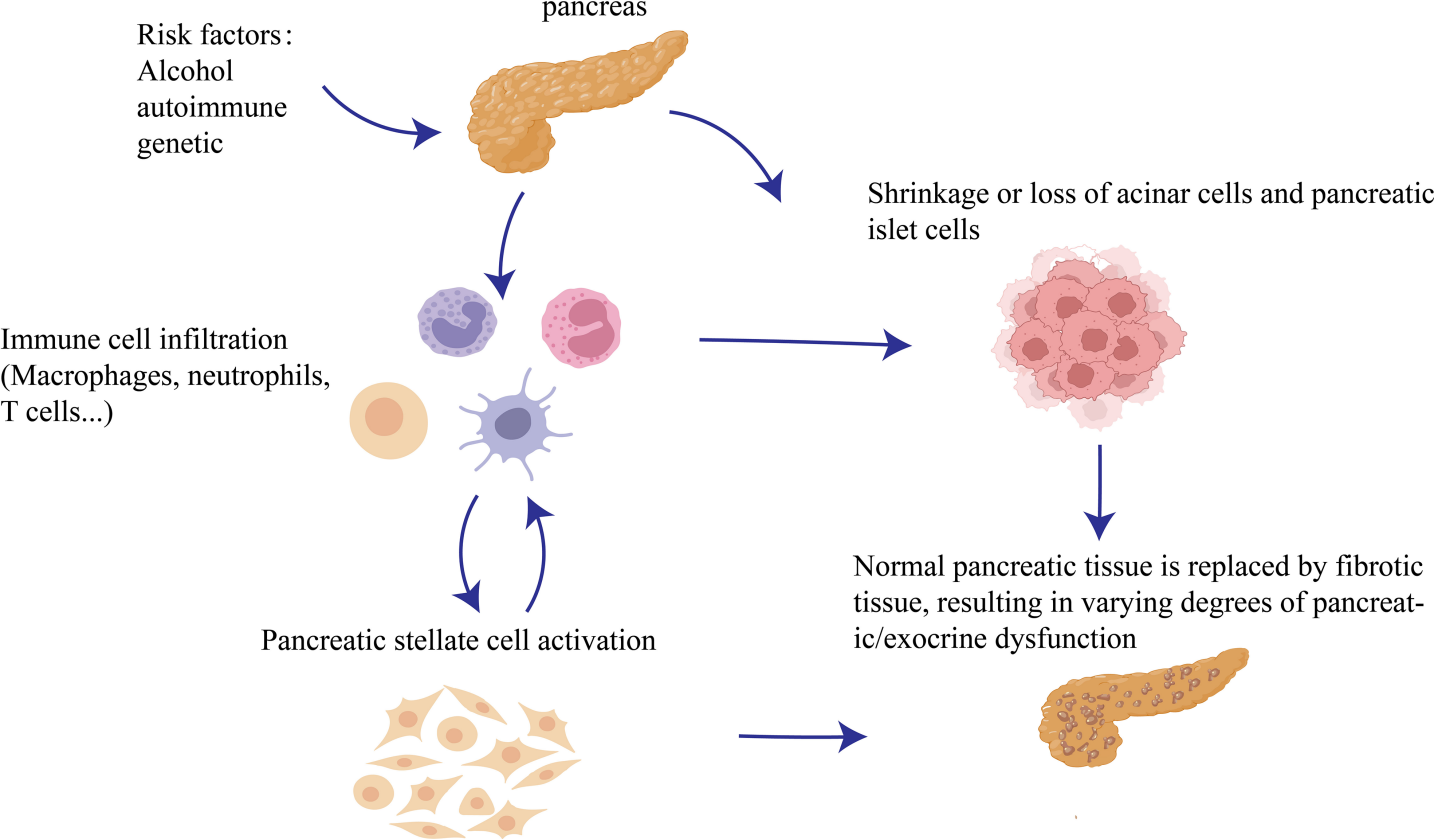
Occurrence of CP: Repeated damage to the pancreatic parenchyma by pathogenic factors will cause more immune cells to recruit into pancreatic tissue and damage acinar cells at the same time, and then PSCs are activated and interact with various immune cells to further aggravate chronic pancreatitis fibrosis.

In recent years, the pathogenesis of chronic pancreatitis has become the focus of people’s attention. A number of studies have shown that the essence of pancreatic fibrosis is the result of excessive deposition of extra cellular matrix(ECM) dominated by collagen fibers in the process of repeated inflammation and necrosis repair of pancreatic tissue, and the degree of activation of PSC in CP patients is positively correlated with the degree of pancreatic tissue fibrosis, ECM synthesis is mainly carried out by PSC, PSC activation plays an important role in the process of chronic fibrosis of the pancreas, and is the target of various triggers. It is the core of pancreatic fibrosis formation (18). The activation of PSCs is regulated by a variety of cytokines and signaling pathways, such as TGF-β/Smad pathway (19), MAPK pathway (20), etc. At the same time, it was found that a variety of immune cells (macrophages, neutrophils, mast cells, eosinophils, T cells, B cells) can also affect the progression of chronic pancreatitis (**Figure 1**).

## 3 The role of immune cells in CP

### 3.1 Innate immune cells

#### 3.1.1 Macrophage in the pancreas and its role in CP

Macrophages can develop from bone marrow hematopoietic stem cells or differentiate from progenitor cells in the early embryo yolk sac stage (21). Fragmentation of damaged cells in the local tissue of the pancreas produces chemotactic stimulants that attract monocytes to pass through the vascular endothelium into the damaged tissue and then transform into macrophages. Macrophages show different phenotypes and functional differentiation under the action of different inducing factors and microenvironments *in vivo*, which is called polarization of macrophages (22). Gordon classified polarized macrophages into two main types: 1) classically activated macrophages (M1), which are induced by interferon γ (IFNγ) and/or lipopolysaccharide (LPS) and characterized by the production of reactive oxygen species and nitrogen substances that play a key role in host defense and anti-tumor immunity; 2) alternatively activated macrophages (M2), after exposure to IL-4/IL-13, are characterized by the expression of the receptor CD206 on the cell surface and by activated macrophages playing a key role in inhibiting inflammation, promoting wound healing, fibrosis, and tumogenesis (23). Not only do macrophages play an important role in CP, M0 macrophages have also been found to be an independent predictor of adverse outcomes in patients with pancreatic ductal adenocarcinoma(PDAC) in patients with PDAC (24).

An increase in macrophages in the pancreas can be observed in models of chronic pancreatitis constructed by different methods. Algul et al. constructed a mouse CP model using cerulein and found many inflammatory cells in the pancreas of mice. Further examination of GR-1 (a protein used as a neutrophil marker) and F4/80 (a protein marker expressed by mature mouse macrophages) revealed that macrophages are the main inflammatory cells infiltrating the pancreas during the course of CP (25). In a dichloride dibutyltin (DBTC)-induced mouse CP model, macrophage infiltration was observed 1 week after modeling and was found to be associated with the progression of pancreatic fibrosis (26). The same Duan et al. found that mice with 20% L-arginine induced CP had significant macrophage infiltration and higher levels of fibrosis, and the development of pancreatic fibrosis in mice with CP was accompanied by a significant increase in F4/80 ^+^ cells in pancreatic tissues, and the level of IL-6 in serum and pancreatic tissues increased. Double-label immunofluorescence staining showed co-localization of IL-6 with F4/80 confirming that infiltrating macrophages during CP are an important source of IL-6 production and that macrophages play a role in the progression of pancreatic fibrosis (27).

To investigate whether the increased macrophages in the CP pancreas are mainly M1 or M2, CD11b monocytes/macrophages were selected for gene expression analysis in a mouse CP model induced by cerulein and further validated by flow cytometry that M2 macrophages, which are mainly anti-inflammatory and promote tissue repair, were predominant in CP (10). In rat pancreatic fibrosis induced by DBTC, immunohistochemistry showed that the number of CD68-expressing M1 macrophages increased at the beginning of pancreatitis and the number of CD163-expressing M2 macrophages was significantly higher in the later stages of pancreatitis, with the appearance of M1 macrophages preceding that of M2 macrophages. Furthermore, double immunofluorescence staining of fibrotic specimens showed that 44% of macrophages co-expressed CD68/CD163 in pancreatic fibrosis, and the results suggest that monocyte-derived CD68 M1 macrophages may be converted to CD163 M2 macrophages during CP. Thus, macrophages may change from M1 phenotype with pro-inflammatory function early in the CP process to M2 phenotype with fibrotic and anti-inflammatory function late in the process (28).

In an *in vitro* assay, Xue et al. co-cultured normal mouse bone marrow-derived macrophages (BMDM) with Transforming growth factor-β1(TGF-β1) stimulated pancreatic stellate cells (PSC) (10). After 48 h, the mRNA expression levels of CD206, IL-13, IL-4, IL-4Rα, TGF-β, platelet-derived growth factor-β (PDGF-β), and other M2-type markers in macrophages were significantly increased, whereas the expression of iNOS in M1-type macrophages was significantly decreased. These results suggest that PSC can polarize macrophages to the M2 type. Meanwhile, this study further explored the relationship between M2-type macrophages and pancreatic fibrosis by inducing CP in IL-4/IL-13 deficient mice, which were less sensitive to CP than wild-type mice. In a CP model constructed in IL-4/IL-13-deficient mice, the expression of α-SMA and Col1a1 related to pancreatic fibrosis decreased, and the expression of CD206 in pancreatic leukocytes decreased, suggesting decreased activation of M2 macrophages. The expression of M2-type macrophages and α-SMA decreased after IL-4/IL-13 blockade and pancreatic fibrosis was significantly alleviated. These results suggested that M2 macrophages play a role in promoting the progression of pancreatic fibrosis. Macrophage interacts with PSC in an autocrine- and paracrine-dependent manner to promote the development of pancreatic fibrosis (10). As the severity of pancreatic fibrosis worsens, the ratio of M2/M1 macrophage cytokines in the pancreas increases (29). Moreover, more M2 macrophages in the pancreas of patients with CP participate in tissue repair, limit the inflammatory response, and interact with PSC to promote pancreatic fibrosis (30). Interestingly, recent analyses have shown that hereditary and idiopathic CP have different immunophenotypes. Although both exhibit M2 polarization and an increased M2/M1 cytokine ratio, idiopathic CP has a higher proportion of CD68^+^ macrophages than hereditary CP (31).

For the pathogenesis of macrophages and chronic pancreatitis, it was found that NF-κB is one of the major players in the recruitment of macrophages to the pancreas and is a star molecule in the pathological immune response to CP. NF-κB is a general term for a group of transcription factors composed of RelA (P65), RelB, c-REL, NF-κB1 (P50/P105), and NF-κB2 (P52/P100). These subunits can be activated by many different stimuli, including bacterial lipopolysaccharide (LPS), viral pathogens, cytokines, or growth factors (32). RelA is a major activator of latent NF-κB enhancers in macrophages (33), and its activation involves the degradation of a family of inhibitory proteins called IκBs. Upon phosphorylation of IκBs by kinases such as the IκB kinase complex in response to various stimuli, IκBs are targeted for degradation by the proteasome, after which the NF-κB subunit is released from the inhibitory complex. NF-κB subunits can then translocate to the nucleus to activate numerous NF-κB-specific target genes (34, 35). NF-κB family members can form multiple combinations of homodimers or heterodimers to activate complex regulatory networks that ultimately activate or repress hundreds of genes (34, 36–38).

**Figure 2:**
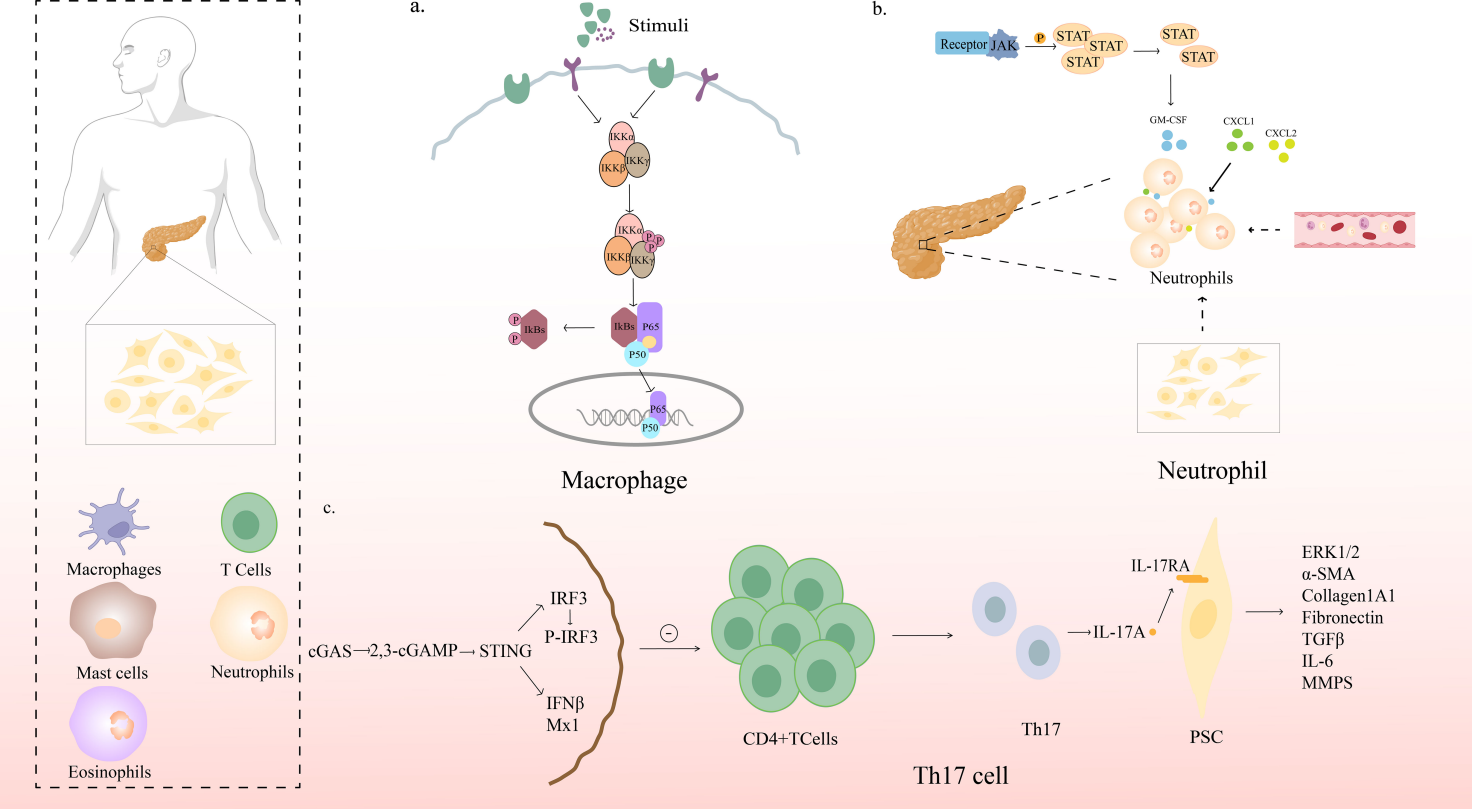
Mechanism of action of immune cells in CP. **(A)** NF-κB signaling pathway in pancreatic macrophages. Upon phosphorylation of IκBs by kinases such as the IκB kinase complex in response to various stimuli, IκBs are targeted for degradation by the proteasome, after which the NF-κB subunit is released from the inhibitory complex; NF-κB subunits can then translocate to the nucleus to activate numerous NF-κB-specific target genes **(B)** STAT5 promotes chronic pancreatitis by enhancing GM-CSF-dependent neutrophil augmentation; **(C)** STING signaling protects against chronic pancreatitis by modulating Th17 response.

The inflammatory targets of NF-κB include cytokines, chemokines, immune receptors, and adhesion molecules (39). Sendler et al. found that macrophages are the main immune cell population migrating to the pancreas during induced pancreatitis in mice, and CD68-positive macrophages were found to phagocytose acinar cell components, including zymogen-containing vesicles, in models of mouse pancreatitis as well as in human necrotic pancreatic tissues. Endocytosed trypsinogen activates macrophages, leading to the translocation of NF-KB and the production of inflammatory cytokines that increase the severity of pancreatitis (40). Sendler et al. also found that damaged acinar cell can release chemokines and inflammatory factors that activate macrophages, which promote the activation of NF-κB subunit P65 and many proinflammatory cytokines such as IL-6, TNF-α, and MCP-1, causing a strong inflammatory response. In conclusion, when the pancreatic tissue is injured, many macrophages are recruited to the damaged site and activate NF-κB to play a role in the progression of CP (**Figure 2A**).

#### 3.1.2 Neutrophils in the pancreas and its role in CP

As neutrophils are usually the first effector cells recruited to sites of inflammation, we usually use neutrophils as a reference marker of pancreatic inflammation by measuring myeloperoxidase (MPO) activity in tissues and reflecting the number of infiltrating neutrophils (6). Although neutrophils do not infiltrate as much as macrophages during CP, they are associated with disease progression and severe disease symptoms (9). Neutrophils can activate various immune and stromal cells to secrete inflammatory cytokines, leading to aggravation of inflammation (40). Neutrophils can also produce large amounts of reactive oxygen species and reactive nitrogen, leading to worsening inflammation (41).

Neutrophils can not only produce a large number of reactive oxygen species and reactive nitrogen, leading to worsening inflammation, but can also activate various immune cells and stromal cells to secrete inflammatory cytokines, leading to aggravation of inflammation (41).

STAT5 is an important member of the signal transducer and activator of transcription (STAT) family and is involved in a wide range of physiological and pathological processes. STAT5 plays a crucial role in lymphocyte development to maintain normal immune system balance (42). STAT is a nuclear transcription factor; however, in resting cells, STAT is present in the cytoplasm. Upon activation, STAT molecules form dimers and enter the nucleus to regulate gene expression. STAT5 can affect cell proliferation, differentiation, survival, and apoptosis, and regulate stem cell self-renewal (42, 43). Previous studies have shown that STAT5 is continuously activated in pancreatic cancer cells (44). Inhibition of STAT5 in pancreatic cancer cells can inhibit tumor growth and metastasis (45) and reduce chemoresistance of pancreatic cancer (44). However, the role of STAT5 in the regulation of inflammation remains poorly understood.

Yuli Lin et al. found that STAT5 is upregulated and activated in CP and that the loss or inhibition of STAT5 alleviates pancreatic inflammation. They further found that the STAT5-induced increase in Granulocyte-macrophage colony-stimulating factor(GM-CSF) expression was responsible for neutrophilia and aggravated pancreatic inflammation (46)(**Figure 2B**).

The chemokine receptor CXCR2 contributes significantly to neutrophil migration *in vivo*. In humans, CXCR2 acts as a receptor for seven different chemokines (CXCL1-3 and CXCL5-8), two of which (CXCL6 and CXCL8) signal through the closely related receptor CXCR1 (47). Mice lack CXCL8 (formerly known as IL-8), and CXCL5 and CXCL6 only have a single orthogonant; therefore, CXCR2 has only five chemokine ligands in mice (48, 49). Steele et al. showed that CXCR2 is essential for the recruitment of neutrophils to the pancreas in models of acute CP and plays a key role in driving the acinar cell damage seen in these models, and that the CXCR2 inhibitory peptide “pepducin” protected wild-type mice from acute CP and significantly reversed established pancreatic inflammation (50). Neutrophil chemokines such as CXCL1 and CXCL2 drive neutrophil recruitment during pancreatic inflammation (41). In summary, we conclude that neutrophils in CP can be recruited to pancreatic tissues by increasing GM-CSF expression induced by STAT5 signaling, and can also migrate to pancreatic tissues under the influence of the chemokine CXCR2, so that neutrophils can participate in CP progression.

#### 3.1.3 Eosinophils in the pancreas and its role in CP

Juniper et al. first reported a significant increase in the peripheral blood eosinophil count in a patient with chronic recurrent pancreatitis with pleural effusion (51). A subsequent study of 122 patients with CP reported significant eosinophilia in 21 patients(17.2%) (52). Another study by Wang showed a high incidence of eosinophilia accompanied by pancreatic ascites and enlargement in patients with CP (53). And Mishra et al. reported for the first time the accumulation of eosinophils in the pancreas and contributed to disease pathogenesis, including fibrosis in cerulein-induced experimental pancreatitis (54), suggesting that eosinophilia may be the cause of pancreatic inflammation and fibrosis progression.

After cerulein-induced acute CP in wild-type mice, anti-MBP immunostaining was performed to confirm the infiltration and accumulation of eosinophils in the pancreas of a cerulein-induced pancreatitis model. The results showed that anti-MBP stained eosinophils and degranulated eosinophil extracellular granules could be detected in the pancreatic tissue sections of cerulein-treated wild-type mice (54). In addition, quantitative real-time PCR and ELISA analysis showed that the levels of IL-5, IL-18, and the chemokines eotaxin-1 and eotaxin-2 transcripts and proteins were significantly increased in cerulein-induced pancreatitis (54).

IL-5 is a well-known factor for eosinophil growth, differentiation, and survival (54, 55) Manohar et al. also demonstrated that endogenous IL-5 deficiency reduced eosinophils in a murine model of cerulein-induced CP, and the induced IL-18 indicated a role for IL-18 in promoting eosinophil accumulation and degranulation during pancreatitis (54). In summary, eosinophils can affect CP through various inflammatory factors (IL-5, IL-18), but research is still lacking.

#### 3.1.4 Mast cells in the pancreas and its role in CP

Mast cells are well-known effector cells that are involved in rapid hypersensitivity reaction (56). Compared to normal pancreatic tissue, the number of mast cells in the CP group was significantly increased, they are located around the residual acinar tissue, suggesting their involvement in pancreatic destruction (57). It has been found that activated mast cells can induce type I collagen expression in mouse skin fibroblasts by secreting TNF-α and TNF-β (58).

AP is a known risk factor for CP progression (59). IL-18 plays an important role in the progression of AP and has been associated with several fibrotic diseases including liver fibrosis (60), cardiac fibrosis (61) and pulmonary fibrosis (62). Therefore, IL-18 may also be involved in the pathogenesis of CP. Lorentz et al. showed that human mast cells can directly secrete IL-18 (63); therefore, they are likely to be involved in pancreatic fibrosis.

### 3.2 Adaptive immune cells

#### 3.2.1 T cells in the pancreas and its role in CP

T cells can be divided into subgroups with different functions, including T helper cells (Th cells), cytotoxic T cells (TC), regulatory T cells (Tregs), memory T cells, natural killer T cells, and γδ T cells (64). Although neutrophils and monocytes/macrophages are the predominant infiltrators of pancreatic inflammation, a local imbalance of T cells in the inflammatory site and circulation has been observed in pancreatitis (9). Nakayama et al. found that severely co-immunodeficient mice developed pancreatitis only after reconstitution with splenocytes, CD4^+^ Tcells, or CD8^+^T cells. Repeated stimulation of the innate immune system is necessary, but not sufficient, to cause pancreatitis, and the involvement of an acquired immune response is essential for the development of the disease (65). A clinical study comparing changes in peripheral immunoactive blood cells during CP and after pancreatic head resection also showed a significant increase in the number of CD3^+^ and circulating CD4^+^ T cells in CP patients (66). These findings suggest that T cells may also play a significant role in the progression of pancreatitis (67, 68). γδT cells and naïve CD4^+^T cells have also been found to be independent predictors of adverse outcomes in PDAC patients in patients with PDAC (24). Nonetheless, little is known about the composition and role of T-cell subsets in pancreatitis (69–71).

Recent studies have shown that CP is associated with disease-specific regulatory T cell responses, and that the pancreatitis-specific IL-10 response is mediated by IL-10^+^IFN-γ FoxP3^+^ regulatory T cells, which are amplified in the blood, bone marrow, and CP lesions. It also has the potential to inhibit the proliferation of autologous conventional T-cells in an antigen-specific manner. Compared with pancreatic cancer, IL-10 concentration increased and IFN-γ levels decreased in CP lesions, suggesting pancreatitis-specific activity of regulatory T cells *in situ (* 70).

James Jupp found a significant increase in the proportion of Th1, Th2, and Th17 cells in the peripheral blood of CP patients. Patients who drank heavily had more Th1 cells than non-drinkers. The infiltrating CD4^+^ T helper cells of the pancreas were mainly Th1 cells (41.5%) and Th17 cells (2.1%), with few Th2 cells (72). Th17 cells produce signature cytokines including IL-21, IL-22, and IL-23. IL-22 plays a protective role in pancreatic inflammation by upregulating the expression of anti-apoptotic genes (Bcl-2 and Bcl-XL), inhibiting the autophagy pathway, and reducing the formation of autophagosomes (73, 74). However, Habtezion’s article published in 2016 showed that activation of AhR can induce IL-22 production, promote experimental CP fibrosis, and neutralize IL-22 to inhibit pancreatic fibrosis (75). IL-17, produced by Th17 (76) and γδ T cells, it is involved in tissue homeostasis, infection (including bacteria and fungi), autoimmunity, cancer progression, and inflammatory disease (77, 78). Overexpression of IL-17A and amplification of γδ T cells in the pancreas aggravates inflammatory immune damage in the pancreas (79). Recent studies have found that STING loss is associated with increased Th17 cell infiltration in the pancreas, and that STING agonists limit this Th17 response. Importantly, anti-IL-17A antibody treatment reduced CP severity in the absence of STING signals. Loss of STING can promote Th17 polarization and PSCs expression of IL-17 receptor by upregulating fibrosis genes. We can conclude that the activation of STING has a protective effect on CP, and that STING signaling regulates the adaptive immune response by reducing the production of IL-17A in CP. It also provided a new therapeutic target for CP (76)(**Figure 2C**).

#### 3.2.2 B lymphocytes in the pancreas and its role in CP

In the last decade, T cells, dendritic cells, and macrophages have been recognized as key regulators of immune response, linking innate and adaptive immunity. Antibody production has long been thought to be determined by T cells; however, recent studies have revealed the existence of many B cell subpopulations with specific regulatory functions capable of modulating T cells and chronic inflammatory responses (80).

Most studies on B cells in pancreatitis are autoimmune pancreatitis (81, 82)However, Simon et al. found that B cells also play a role in the pathogenesis of CP; and in the context of pancreatitis, B cells inhibit pancreatic regeneration, and their transport and/or function is regulated by hypoxia and HIF1α (83). Importantly, targeting B cells with αCD20 monoclonal antibodies or Bruton tyrosine kinase (BTK) inhibitors offers a potential therapeutic approach for the treatment of pancreatitis. Moreover, the BTK inhibitor ibrutinib improves survival in mouse models of pancreatic cancer (84, 85), Therefore, the B-cell targeting approach may be beneficial for both pancreatitis and pancreatic cancer.

## 4 Interaction between immune cells and pancreatic stellate cells affects the progression of chronic pancreatitis

### 4.1 Pancreatic stellate cells

Pancreatic stellate cells (PSCs) are pluripotent effector cells located around the acinar cell, small blood vessels and pancreatic ducts (86). PSCs constitute a small fraction of all pancreatic cells under physiological conditions but are essential for maintaining normal pancreatic architecture. Quiescent PSCs are characterized by vitamin A-rich lipid droplets; however, when PSCs are activated by proinflammatory substances released by infiltrating immune cells around the pancreas, these perinuclear lipid droplets disappear from the cytoplasm and become a myofibroblast-like phenotype, expressing the activation marker alpha smooth muscle actin, which gradually replaces healthy pancreatic tissue and eventually develops pancreatic fibrosis (13).

Physiologically, transformation of quiescent PSCs into a proliferating myofibroblast-like phenotype is an autonomous repair response to tissue damage (86). Aberrant activation of PSCs is a major pathological feature of fibrosis in CP (86, 87). Recent *in vitro and in vivo* studies have demonstrated that activated PSCs play a central role in CP-related fibrosis by regulating the synthesis and degradation of ECM proteins such as tissue inhibitors, matrix metalloproteinases (TIMPs), and metalloproteinases (MMPs) (88–90). Other studies have also found that in addition to regulating ECM, PSCs can secrete acetylcholine, which may act as an intermediate regulator of cholecystokinin-mediated pancreatic exocrine secretion (91). Repeated and persistent pancreatic injury and inflammation are key to the initiation of fibrosis. Several *in vitro* studies have demonstrated that pancreatic injury and inflammation can expose PSCs to a variety of cytokines, such as IL-1, IL-6, TNF-α, PDGF, TGF-β1, activin A, ethanol, and its metabolites, which can act as regulators of PSCs activation, causing oxidative stress and extensive changes in the composition of the ECM (92).

### 4.2 Interaction between immune cells and pancreatic stellate cells

Chanjuan Shi et al. co-cultured static IPS-1 cells (an immortem PSC cell line) with two different macrophage cell lines RAW264.7 and BMDM-WT and found that when static IPS-1 cells were co-cultured with RAW264.7 macrophage derived cell lines, IPS-1 is activated. IPS-1 cells did not exhibit these morphological changes in the absence of macrophage co-culture, suggesting that macrophages activate PSC (93).

To determine whether the factors released by PSC can change the activation and polarization state of pancreatic macrophages, some studies have co-cultured PSC and BMDMs *in vitro*. Co-culture with activated PSC showed M2 characteristics, indicating that PSC release factors promoted the polarization of macrophages toward M2 (10). However, it has also been shown that IPS-1 cells only alter macrophage cytokine production and do not induce a definite M1 or M2 phenotype (93).

**Figure 3:**
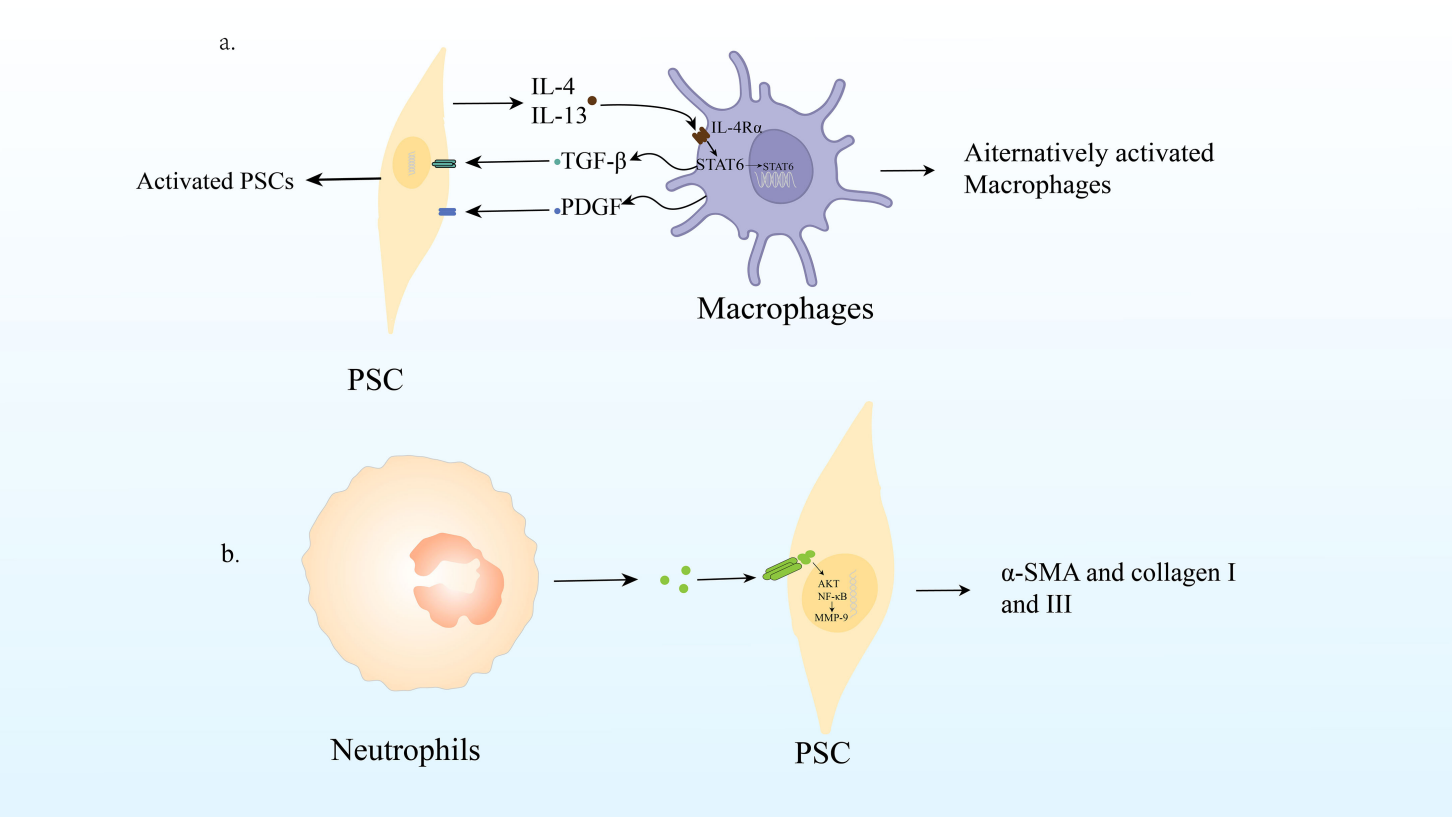
**(A)** Macrophage infiltration and polarization lead to PSC activation and promote CP. **(B)** Neutrophil production of ROS can lead PSC to activate signaling pathways AKT and NF- B, up-regulate MMP-9 and Twist, produce α-SMA, collagen I and III, leading to the fibrosis process of CP.

As mentioned above, PSC can interact with macrophages to promote the occurrence of CP, which is the mechanism underlying their interaction. Studies have been conducted to understand the mechanisms by which macrophage and hepatic stellate cell (HSC) activation interact using cytokines produced by cells cultured alone or together. They found that quiescent IPS-1 cells not only produced high levels of IL-6 and MCP-1, two chemokines known to act on macrophages (94), KC/CXCL1 was also produced. RAW264.7 cells alone produced low levels of cytokines; however, co-culture with IPS-1 cells significantly increased the levels of cytokines, especially granulocyte colony stimulating factor(G-CSF)/CSF3, MCP-1, macrophage inflammatory protein 1α (MIP-1α)/CCL3, MIP-1β/CCL4, MIP2/CXCL2, and TNF. As the cells were cultured together, there is no way to know which cell types express which cytokines; however, the production of CCL3, CXCL2, and TNF is commonly associated with macrophages (93). Previous studies have shown that TGF-β and PDGF produced by macrophages are effective activators of PSC (89). PSC were treated with TGF-β and PDGF-β, and IL-4/IL-13 expression was examined. Interestingly, this study showed that PDGF, especially TGF-β, could induce both αSMA and IL-4 and IL-13 genes in PSC. These results indicated that PSC promoted the activation of macrophages in an IL-4Rα signal-dependent manner, and macrophages had the ability to induce PSC activation and produce IL-4R ligand (10)(**Figure 3A**).

Recent studies have shown that reactive oxygen species (ROS) produced by neutrophil infiltration are important regulators of the pathogenesis and development of pancreatitis. Pancreatic fibrosis is known to result from a dynamic cascade mechanism that begins with acinar cell injury and necrosis, followed by inflammation, macrophage activation, platelet aggregation, release of growth factors and ROS, PSC activation, stimulation of extracellular matrix synthesis, and decreased matrix degradation (95).

Neutrophils play a fundamental role in the innate immune response. Under sufficient stimulation, neutrophils release excessive ROS, which can induce cellular and tissue damage (96). In CP, ROS can activate downstream redox-sensitive signaling pathways AKT and NF-ĸB in PSC, upregulate MMP-9 and Twist, produce α-SMA and collagen I and III, leading to the fibrosis process of CP (97). Recent studies have shown that antioxidants reduce islet fibrosis in animal models of type 2 diabetes *in vitro* and *in vivo (* 98). PSC shares homology with HSC (95). De Bleser et al. also demonstrated that ROS stimulates collagen I production in aHSCs/myofibrils in hepatitis, acting as an intracellular signaling mediator of TGF-β1-induced fibrosis (99). All these directly or indirectly indicates that ROS play an important role in CP fibrosis (**Figure 3B**).

In addition to the most studied macrophages and neutrophils, stellate cells have also been found to interact with other immune cells. For example, in liver-related diseases, interactions between HSCs and other immune populations are less extensively catalogued but important. HSC activation in experimental fibrosis models is reduced in immunodeficient (SCID) mice and rescued by adoptive transfer of lymphocytes, especially CD8^+^T cells (100). Similarly, B cells and HSCs form a pro-fibrogenic network in mice subjected to ${\text{CCl}}_{4}^{+}$ liver injury. Retinoic acid (RA) signaling by HSCs promotes B cell survival and activation while, in turn, B cells secrete inflammatory cytokines (101). Retinoic acid signaling has emerged as an important immunomodulatory mediator, particularly in promoting Th17 cell differentiation. RA receptor (RAR) synthetic agonists and all-trans retinoic acid (ATRA) have also shown direct anti-fibrotic effects on HSCs (102, 103). Similarly, interferon-γ (IFNγ), produced by many immune cells including NK, NKT and T cells, has direct anti-fibrogenic activity on HSCs (104–106).

By analogy with liver fibrosis disease, we speculate that immune cells other than macrophages and neutrophils may also interact with PSCs to affect chronic pancreatitis, but there are no definitive studies to prove how they interact with each other.

## 5 Targeting pancreatic stellate cells and immune cells for CP

### 5.1 Targeting pancreatic stellate cells for CP

It is well known that PSC are closely related to the fibrosis of CP, so drug therapy targeting pancreatic stellate cells may be an effective way to treat CP pancreatic fibrosis.

Schwer et al. proved, for the first time, that curcumin inactivated PSCs by inhibiting their proliferation. This is achieved by reducing extracellular signal-regulated protein kinases 1 and 2 (ERK1/2) activation with heme oxygenase-1(HO-1), which increases cell carbon monoxide levels and activates mitogen-activated protein kinase (MAPK) P38, resulting in reduced PSC proliferation (107). Curcumin and the three phenolic compounds significantly inhibited the mRNA and protein levels of α-SMA, type I collagen, fibronectin, and other fibrosis mediators in TGF-β-activated PSCs, and the mechanism was related to the downregulation of the NF-κB signaling pathway. These results suggest that curcumin may be an anti-fibrotic agent for the treatment of pancreatic fibrosis, including PDAC (108). Recently, a new synthetic curcumin analog (L49H37) was discovered and compared with traditional curcumin. It was found that L49H37 induced PSCs apoptosis at a concentration 10 times lower than curcumin (109).

Tsang et al. demonstrated that treatment with rhein (50 mg/kg/day) in a cerulein-induced CP mouse model significantly attenuated fibrosis by reducing the immune reactivity of fibrotic activators (including α-SMA and TGF-β) in pancreatic tissues. It then reduces fibronectin (FN1) and type 1 collagen deposition in the exocrine (110). It also inhibited the expression of sonic hedgehog signal (SHH, which is positively correlated with the degree of cyanin-induced fibrosis) and GLI1 in the pancreatic tissues of CP mouse models. In cultured PSCs, 10 μM rhein significantly inhibited the expression of TGF-β-stimulated fibrosis markers α-SMA, FN1, COL I-α1, and SHH (110).

Several studies using the CP model have assessed the anti-fibrotic effects of ellagic acid. Ellagic acid treatment significantly inhibits the development of pancreatic fibrosis in experimental CP models in Wistar Bonn/Kodori rats. The mRNA expression of α-SMA and TGF-β1 was significantly decreased. In addition, ellagic acid treatment significantly reduced macrophage and monocyte infiltration as well as *in vitro* ROS production by PSCs (111). Moreover, ellagic acid inhibited PSC proliferation and migration of PDGF-BB. Ellagic acid reduces the activation of downstream signaling pathways of the RAF proto-oncogene serine/threonine protein kinase (C-Raf)/MAPK/ERK and PI3K/AKT, which are important for PSC proliferation and migration (112). Further studies showed that ellagic acid inactivated PSC by attenuating α-SMA and ECM procollagen type I and III protein levels. In addition, ellagic acid treatment inhibited IL-1β- and TNF-α-induced MCP-1 in AP-1 associated PSC but did not inhibit NF-κB. They also found that ellagic acid prevented PSC from transitioning from a resting state to a myofibroblast-like phenotype (112).

Isoliquiritigenin (ILG) is a chalcone-type dietary compound extracted from Glycyrrhiza. The effects of ILG on pancreatic fibrosis and inflammation were studied using a murine model of cerulein. The results showed that ILG significantly reduced pancreatic fibrosis and macrophage infiltration. Further *in vitro* studies of human pancreatic stellate cells (hPSCs) showed that ILG inhibited the proliferation and activation of hPSCs, possibly because ILG negatively regulates the activity of ERK1/2 and JNK1/2. In addition, ILG significantly inhibited RAW 264.7 macrophage polarization to an M1 phenotype by attenuating NF-κB, whereas polarization to an M2 phenotype was almost unaffected. These results suggest that ILG may be a potential anti-inflammatory and antifibrotic agent for CP treatment (113) (**Table 1**).

**Table 1:** Effects of drugs on pancreatic stellate cells and their action pathways.

Drug	Role	Mechanism	Model	Reference
**Curcumin**	Proliferation of PSC	Activation of ERK1/2, MAPK, TGF-β, NF-KB	Male Wistar rat	(107, 108)
**L49H37**	Apoptosis of PSC	Cut P21WAF1/Cip1		(109)
**Rhein**	Activation of PSC	Cut TGF-β-stimulated fibrosis markers α-SMA, FN1 and COL I-α1 as well as SHH was decreased		(110)
**Ellagic Acid**	Proliferation and Migration of PSC	Cut PDGF-BB, α-SMA and TGF-β1 mRNA expression	Experimental CP model in WistarBonn/Kodori rats	(111, 112)
**ILG**	Proliferation and Activation of PSC	Inhibition of ERK1/2, JNK1/2 and NF-κB	Mouse model of CP	(113)

### 5.2 Targeting macrophages for CP

In CP, macrophages interact with PSCs by secreting various cytokines to create an inflammatory microenvironment conducive to pancreatic fibrosis, thus aggravating pancreatic injury. Therefore, drug therapy targeting macrophages may be an appropriate treatment for CP pancreatic fibrosis.

Berberine (BR), an activator of AMP-activated protein kinase (AMPK), has antioxidant and anti-inflammatory properties. BR inhibits TGF-β/Smad signaling and M2 macrophage polarization in an AMPK-dependent manner. Sapana et al. investigated the effect of BR on cerulein-induced CP (CP). The results showed that BR treatment (10 mg/kg) significantly reduced oxidative stress, histological changes, inflammatory cell infiltration, and collagen deposition in pancreatic tissue. BR treatment also prevented cerulein-induced activation of pancreatic astrocytes (PSCs) and extracellular matrix (ECM) deposition by downregulating the expression of α-SMA, collagen 1a, collagen 3a, and fibronectin. BR significantly activated the AMPK signaling pathway compared to cerulein-challenged mice. Additionally, BR inhibited TGF-β/Smad signaling and macrophage polarization in an *in vivo* model of cerulein-induced CP. In conclusion, the results strongly suggest that BR treatment protects cerulein-challenged mice with CP and associated fibrosis progression by inhibiting TGF-β1/Smad signaling and AMPK-dependent polarization of M2 macrophages (114).

Dasatinib significantly improved pancreatic fibrosis and macrophage infiltration in the CP mouse models. Further RNA-seq and *in vitro* validation analyses showed that dasatinib significantly inhibited the proliferation and activation of PSCs through the TKs/GSK3β/βcatenin pathway. In addition, dasatinib significantly inhibited M1 and M2 polarization of macrophages and hindered their recruitment and crosstalk with PSC. In conclusion, dasatinib may be a potential treatment for CP because of its antifibrotic and anti-inflammatory properties (115).

One study randomly divided mice into control, CP, and Dachaihu decoction (DCHD) groups. Mice in the CP and DCHD groups were intraperitoneally injected with 20% L-arginine. Mice in DCHD group were given DCHD intragastric administration after CP induction for 1 week CP mice induced by 20% L-arginine showed significant macrophage infiltration and higher levels of fibrosis than the control group. The serum IL-6 concentration was significantly higher. Dual immunofluorescence staining revealed that IL-6 and F4/80 were co-expressed in the pancreas. After DCHD treatment, the infiltration of macrophages and degree of fibrosis in the pancreas were significantly reduced, IL-6, MCP-1, MIP-1α mRNA, and fibronectin levels decreased. Therefore, it was concluded that the leading role of macrophages in CP development is mainly related to the production of IL-6. DCHD can effectively improve pancreatic fibrosis by inhibiting the infiltration of pancreatic macrophages and the secretion of inflammatory factors (27).

Sulindac is an aryl chain acid non-steroidal anti-inflammatory drug that is reversibly converted to sulindac sulfide, an anti-inflammatory active compound in the body that inhibits COX-1 and COX-2 activities, reduces prostaglandin(PG) synthesis, and exerts analgesic and anti-inflammatory effects (116). Inhibition of CP by Sulindac was achieved by significantly reducing infiltration of myeloperoxidase-positive neutrophils and Mac-3-positive macrophages in the pancreas, and sulindac treatment also significantly reduced TNF-α and MCP-1 mRNA expression in the pancreas (117). This may be one of the key mechanisms underlying sulforaphane-mediated chemoprevention of pancreatic inflammation and fibrosis.

Baicalin is a flavonoid extracted from the roots of Scutellaria baicalensis Georgi, a dicotyledonous plant of the Labiatae family with a wide range of pharmacological effects, including anti-inflammatory and oxygen radical scavenging effects (118). PSC supernatants containing high concentrations of MCP-1 enhanced the migratory ability of BMDM, and the expression of MCP-1 and F4/80 was significantly reduced in the pancreas of baicalin-treated CP mice, indicating that baicalin could inhibit macrophage migration by reducing the level of MCP-1 in the supernatant of PSCs, which was caused by the inhibition of TGF-β-activated kinase 1, TGF-β1, TGF-βR1, NF-κB, and other signaling pathways in PSCs (119) (**Table 2**).

**Table 2:** Effects of drugs on Macrophages and their action pathways.

Drug	Role	Mechanism	Model	Reference
**Berberine (BR)**	Polarization of macrophages	Inhibition of TGF-β/Smad signal and activation of AMPK signal	Mouse model of CP	(114)
**Dasatinib**	Infiltration of macrophages	The TKs/GSK3 beta/beta catenin way; M1 and M2 polarization of macrophages were inhibited	Mouse model of CP	(115)
**DCHC**	Infiltration of macrophages	The mRNA levels of IL-6, MCP-1, MIP-1α and fibronectin were decreased	Mouse model of CP	(27)
**Sulindac**	Inhibition of macrophage infiltration	Reduced expression of TNF-α and MCP-1 mRNA in the pancreas	Mouse model of CP	(116, 117)
**Baicalin**	Inhibition of macrophage migration	Inhibition of TGF-β-activated kinase 1, TGF-β1, TGF-βR1, NF-κB and other signaling pathways in PSCs	Mouse model of CP	(118, 119)

### 5.3 Targeting other immune cells for CP

The above mentions that immune cells influence the progression of chronic pancreatitis. Due to their key role in inflammation, many studies have found that immune cells other than macrophages are considered potential therapeutic targets for chronic pancreatitis. We explore here the possibility of targeting immune cells to treat chronic pancreatitis.

The pathological changes in CP depend on the recruitment of innate immune cells to the site of initial tissue injury and the coordination of downstream inflammatory pathways. The chemokine receptor CXCR2 drives neutrophil recruitment during inflammation, and Jennifer et al. tested the therapeutic potential of CXCR2 inhibition in the prevention and treatment of chronic pancreatitis. They used CXCR2-inhibiting peptidase in a wild-type mouse model of chronic pancreatitis for full treatment. The pancreas of CXCR2 peptidase-treated mice was significantly protected and far fewer apoptotic cells were present. Peptidase therapy also reduced the infiltration of the pancreas by MPO neutrophils and F4/80 monocytes (50). This finding found that CXCR2 suppression does protect the pancreas from chronic pancreatic inflammation, and given that CXCR2 inhibitors are now available in the clinic (120), we believe these data support future CXCR2-based therapeutic interventions.

Metalloproteinase 17 (ADAM17) mediates the inflammatory response by releasing biologically active inflammatory cytokines and mediators, including tumor necrosis factor α (TNFα) and soluble IL-6 receptor (sIL-6R), which drives trans-signaling of pro-inflammatory IL-6. However, the role of ADAM17 in pancreatitis is unclear. To investigate whether blocking ADAM17 affects the progression of chronic pancreatitis, Brendan et al. constructed a model of chronic pancreatitis using Adam17ex/ex mice (which are homozygous Adam17ex alleles that result in a significant decrease in ADAM17 expression) and their wild-type (WT) homogeneous mice. ADAM17 expression upregulated in pancreatic tissues in animal models of pancreatitis. In addition, genes targeting ADAM17 (Adam17ex/ex mice) and treatments (ADAM17 predomain inhibitors [A17pro]) improved experimental pancreatitis, which was associated with a reduction in the IL-6 transsignaling/STAT3 axis. This leads to a decrease in inflammatory cell infiltration, including T cells and neutrophils, and a decrease in pancreatic necrosis and fibrosis. In addition, upregulation of the ADAM17/IL-6 transsignal/STAT3 axis is a feature in patients with pancreatitis. Taken together, our findings suggest that ADAM17 protease plays a key role in the pathogenesis of pancreatitis, which may provide ideas for designing new treatment options to combat pancreatitis (121).

The macrolide immunosuppressant tacrolimus, also known as FK-506 or its trade names Prograf and Advagraf, binds to FKBP-12 (an immunophilin responsible for signal transduction) and then forms a complex with Ca2^+^, calmodulin, and calcineurin to suppress the action of nuclear factor of the activated T cells (122). Thus, tacrolimus is a topical inhibitor of calcineurin, a protein phosphatase that is essential for the T-cell activation (123). A recent study has demonstrated that tacrolimus has a preventive effect on CP in male Wistar Bonn/Kobori rats through inhibition of the acinar cell apoptosis and abnormal infiltration of CD4^+^ and CD8^+^ T cells. Additionally, FK506 effectively suppressed the development of AIP through augmentation of the infiltrated T-cell apoptosis by inhibiting Bcl-2; but not Bax (124). However, Ito et al. found that FK-506 could induce AP at therapeutic doses *via* elevation of an abnormal secretion of the pancreatic enzymes when the pancreas is overstimulated (125). Together, these findings indicate that tacrolimus can be selectively used for CP and AIP, but not for AP (**Table 3**).

**Table 3:** Effects of drugs on other Immune Cells and their action pathway.

Drug	Role	Mechanism	Model	Reference
**CXCR2-inhibiting peptidase**	Infiltration of neutrophils	Inhibits neutrophil recruitment	Mouse model of CP	(50)
**A17pro**	Reduced IL-6 trans signaling/STAT3 axis	Reduced T cell and neutrophil infiltration	Mouse model of CP	(121)
**Tacrolimus**	Local inhibitors of calcineurin; Inhibits Bcl-2	Inhibits acinar apoptosis and abnormal infiltration of CD4^+^ and CD8^+^ T cells, enhances infiltrating T cell apoptosis	Mouse model of CP	(124)

## 6 Conclusions and future perspectives

CP is the result of a combination of factors, including multiple precipitating factors and complex pathogenesis. Regardless of the cause of CP, immune cells play an important role in the pathogenesis and severity of the disease. In this review, we highlight various immune cells and the role of PSCs in CP.

PSC activation plays an important role in the process of chronic fibrosis of the pancreas, is the target of various causative effects, and is the core of pancreatic fibrosis formation, so drug therapy targeting pancreatic stellate cells may be an effective way to treat CP pancreatic fibrosis. Here we also summarize the drugs that can target PSC for the treatment of CP found so far, including Curcumin, L49H37, Rhein, Ellagic Acid, ILG.

For immune cells, a variety of immune cells are involved in influencing CP. Among them, macrophages are the most studied immune cells in CP, and they are also the most important immune cells. *In vivo* and *in vitro* experiments can prove that macrophages promote pancreatic fibrosis in CP, and can also affect the progression of CP by interacting with PSCs. In addition to macrophages, we also summarized the role of remaining immune cells in CP, for example, in innate immune cells, not only neutrophils are recruited into pancreatic tissue by CXCR2, causing pancreatitis to worsen; mast cells and eosinophils are also involved in the pathogenesis of CP; At the same time, adaptive immune cells (T cells and B cells) are also involved in the pathogenesis of CP. In recent years, targeted therapy for these immune cells has become a feasible direction for the study of CP, and it has been found that Berberine, Dasatinib, DCHC, Sulindac, and Baicalin can act on macrophages to alleviate CP.

Current treatment of CP is aimed at controlling pain and addressing exocrine and endocrine dysfunction. However, with the in-depth exploration of the pathogenesis of CP, a clearer understanding of the pathogenesis of CP and changes in the immune microenvironment will provide richer ideas for the treatment of CP and open up broader treatment prospects. The direction of our future efforts should also focus on exploring the various immune cells involved in CP and the various interactions between them, so as to provide the possibility of curing CP; To explore the relationship between the nervous system and the onset of CP pain to improve the quality of life of patients.

## Author contributions

YZ, W-QZ, and X-YLiu reviewed literature and originally drafted the manuscript. QZ, and TM contributed to editing and embellished the manuscript. X-YLi approved the final version of the manuscript. All authors contributed to the article and approved the submitted version.
